# Molecular mapping of a core transcriptional signature of microglia-specific genes in schizophrenia

**DOI:** 10.1038/s41398-023-02677-y

**Published:** 2023-12-13

**Authors:** Anna M. Fiorito, Eric Fakra, Guillaume Sescousse, El Chérif Ibrahim, Romain Rey

**Affiliations:** 1 Lyon Neuroscience Research Center, INSERM U1028, CNRS UMR 5292, PSYR2 Team, University of Lyon, Lyon, France; 2 Centre Hospitalier Le Vinatier, Bron, France; 3 Department of Psychiatry, University Hospital of Saint-Etienne, Saint-Etienne, France; 4 Aix-Marseille Univ, CNRS, INT, Institut de Neurosciences de la Timone, Marseille, France; 5 Fondation FondaMental, Créteil, France

**Keywords:** Molecular neuroscience, Schizophrenia

## Abstract

Besides playing a central role in neuroinflammation, microglia regulate synaptic development and is involved in plasticity. Converging lines of evidence suggest that these different processes play a critical role in schizophrenia. Furthermore, previous studies reported altered transcription of microglia genes in schizophrenia, while microglia itself seems to be involved in the etiopathology of the disease. However, the regional specificity of these brain transcriptional abnormalities remains unclear. Moreover, it is unknown whether brain and peripheral expression of microglia genes are related. Thus, we investigated the expression of a pre-registered list of 10 genes from a core signature of human microglia both at brain and peripheral levels. We included 9 independent Gene Expression Omnibus datasets (764 samples obtained from 266 individuals with schizophrenia and 237 healthy controls) from 8 different brain regions and 3 peripheral tissues. We report evidence of a widespread transcriptional alteration of microglia genes both in brain tissues (we observed a decreased expression in the cerebellum, associative striatum, hippocampus, and parietal cortex of individuals with schizophrenia compared with healthy controls) and whole blood (characterized by a mixed altered expression pattern). Our results suggest that brain underexpression of microglia genes may represent a candidate transcriptional signature for schizophrenia. Moreover, the dual brain-whole blood transcriptional alterations of microglia/macrophage genes identified support the model of schizophrenia as a whole-body disorder and lend weight to the use of blood samples as a potential source of biological peripheral biomarkers.

## Introduction

Schizophrenia (SZ) is a complex polygenic disorder [1] whose onset typically arises during adolescence. Over the past decade, results from genome-wide association studies have allowed researchers to identify hundreds of variants associated with the SZ risk, shedding light on SZ pathogenesis. Indeed, the interaction of multiple genetic factors with each other and with environmental risk factors is thought to result in pathological neurodevelopmental processes underlying SZ. To date, the strongest genetic association involves the Major Histocompatibility Complex locus [2, 3]. In humans, this locus is essentially related to immune function.

Accordingly, the role of immunity and inflammation in SZ is now largely recognized [4], while microglia, the resident immune cells of the central nervous system, has raised increasing interest [5]. Remarkably, microglia are not only involved in immunity and inflammation but are also required by the developing brain for critical neurodevelopmental processes such as synaptic pruning [6, 7] or neurogenesis regulation [8] which have been involved in the pathophysiology of SZ [9–12].

In this light, some studies have looked for more direct evidence of microglia dysfunction in individuals with SZ, using techniques such as translocator protein (TSPO) positron emission tomography (PET) (microglia activation) and immunohistochemistry in postmortem samples (microglia morphology and density). However, various meta-analyses summarizing these studies reported inconsistent results [13–16]. Poor cellular type specificity or insufficient sensitivity may explain the TSPO-PET heterogeneous results [17], while microglia density and morphology are too simplistic parameters which only offer a partial picture of microglia functioning.

Microglia function has also been explored by gene expression studies which are more specific and more likely to accurately reflect the complex nature of microglial functioning. A meta-analysis of such studies in human postmortem tissues obtained from various brain regions found an overall decreased microglia gene expression in individuals with SZ compared with healthy controls (HCs) [15]. Although supporting the dysregulation of microglia in SZ, Snijders et al.‘s results have three major limits. Firstly, the meta-analytic results were pooled from various brain regions precluding finer conclusions regarding a potential region heterogeneity of microglia alterations in SZ. Secondly, the original studies included in the meta-analytic work didn’t explore the same candidate genes across included studies, thus preventing meta-analytic results at the gene level. Finally, presence of a publication bias was confirmed by Egger’s test, suggesting the meta-analysis was biased towards positive results.

In light of these limits, more studies are needed to replicate and extend current knowledge regarding dysregulation of microglia gene expression in individuals with SZ. Such studies should systematically explore the same set of candidate microglia-specific genes in various brain regions of individuals with SZ compared with HCs, allowing the identification of abnormal transcription at the region level. Furthermore, while postmortem studies allow the investigation of SZ pathogenesis through direct access to brain tissues, there is a dire need for readily accessible SZ biomarkers. In this regard, peripheral tissues are a promising method to probe the transcriptome in a minimally invasive way and allow for the identification of biological peripheral biomarkers of SZ disorder [18]. Noteworthily, in SZ, blood-based microarray meta- [19] and mega- [20] analyses revealed differentially expressed genes involved in immunologic functions, including microglia-related genes.

Using Gene Expression Omnibus (GEO) datasets, in this study we aimed to explore the expression of microglia genes at the central and peripheral levels in individuals with SZ compared with HCs. Notably, the present study takes into account the various limitations of current literature regarding microglia gene expression studies in SZ. Especially, we systematically explored in 8 different brain regions and 3 peripheral tissues a set of 10 genes selected through a comprehensive approach and belonging to a core transcriptional signature of human microglia [21, 22]. There was no overlap between the data used in the present study and that from Snijders et al.’s meta-analysis [15], allowing us to produce new results replicating, refining and expanding current knowledge regarding SZ-associated microglia alterations.

## Material and methods

Data collection, selection of candidate microglia genes, hypotheses, and statistical analyses were pre-registered on AsPredicted.org (#*67610*, https://aspredicted.org/285rn.pdf), on March 6th, 2021. It should be noted that datasets included in this study were slightly different from those pre-registered (see Supplementary Methods for further details on this deviation from the pre-registration).

### Inclusion of genes from a core human signature of microglia

**Fig. 1:**
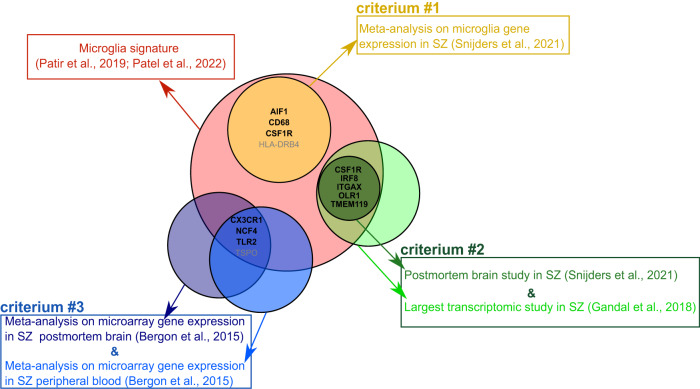
Selection of candidate genes. Venn diagram depicting the procedure of selection of candidate genes. Only genes that were part of a core transcriptional signature of human microglia [21, 22] were included. Based on previous work, we identified microglia genes that seems to be altered in schizophrenia (SZ) according to three criteria. Firstly, we selected all the genes differentially expressed in individuals with SZ compared with healthy controls (HCs) in at least one of the studies included in the meta-analysis of Snijders et al. [15] (criterium #1). Secondly, we selected genes differentially expressed both in a postmortem study exploring candidate genes [15] and in the largest transcriptomic study to this day in SZ [100], as reported in Snijders et al. [15] (criterium #2). Thirdly, we selected genes that were consistently differentially expressed in individuals with SZ compared with HCs in a meta-analysis including results from postmortem brain and peripheral tissues samples [19] (criterium #3). Included genes are noted in black bold font.

To limit the number of multiple comparisons (associated with an increase of Type I errors) and the subsequent lack of power associated with false discovery rate corrections, we limited the number of candidate genes, which were selected using a comprehensive approach. See Fig. 1 for details on selection of candidate genes.

### Search and inclusion criteria of dataset

Datasets were searched through GEO database, a public repository which archives and freely distributes genomic data from postmortem brain or peripheral tissues samples obtained from HCs and patients suffering from various disorders [23]. With the aim to identify altered expression of microglia genes both at the central and peripheral level in individuals with SZ, we explored the GEO database for datasets providing gene expression results from individuals with SZ and HCs.

We included datasets compliant with data extraction through shinyGEO [24], an application implemented using R [https://www.r-project.org/], shiny [http://shiny.rstudio.com/] and GEOquery package [25]. Using GEO database, we therefore searched for studies on SZ, using homo sapiens as the primary organism, and filtering by gene series and microarray technology.

We included datasets obtained from original studies using samples from SZ and HC individuals, and providing microarray-derived gene expression results acquired from postmortem brain samples (not restricted to the neuronal cell population) or peripheral tissues samples.

### Demographic characteristics and quality data

For each GEO dataset, the quality data of the samples (i.e., RNA integrity number (RIN), brain pH, postmortem interval) and the demographic characteristics (i.e., age and sex) of the subjects who provided brain or peripheral tissues are summarized in Tables 1–2.

**Table 1:** Demographic and quality characteristics of the postmortem brain samples.

Brain region	Subjects (*n*)	Age (mean ± SD)			Sex (M/F)			Postmortem interval (mean ± SD)			brain pH (mean ± SD)			RIN (mean ± SD)		
		HC	SZ	*p*-value^a^	HC	SZ	*p*-value^a^	HC	SZ	*p*-value^a^	HC	SZ	*p*-value^a^	HC	SZ	*p*-value^a^
Hippocampus GSE53987	18 HC, 15 SZ	48.16 ± 10.95	45.73 ± 8.79	0.493	9/9	9/6	0.729	19.39 ± 5.2	19.4 ± 7.2	0.994	6.61 ± 0.21	6.43 ± 0.31	0.065	7.37 ± 0.64	6.54 ± 0.52	<0.001*
Associative striatum GSE53987	18 HC, 18 SZ	48.44 ± 10.82	45 ± 8.75	0.301	10/8	10/8	1	19.75 ± 5.14	19.9 ± 7.07	0.945	6.59 ± 0.23	6.47 ± 0.37	0.233	8.21 ± 0.69	7.87 ± 0.80	0.182
Anterior PFC GSE17612	23 HC, 28 SZ	69.04 ± 21.55	73.32 ± 15.2	0.776	11/11 + 1 NA	19/9	0.251	9.9 ± 4.39	8.71 ± 6.98	0.058	6.5 ± 0.29	6.15 ± 0.21	0.001*	-	-	-
Cerebellum GSE35974	50 HC, 44 SZ	45.8 ± 9.33	43.18 ± 9.53	0.208	31/19	32/12	0.283	27.58 ± 11.13	33.27 ± 15.45	0.033*	6.47 ± 0.32	6.43 ± 0.25	0.297	-	-	-
Parietal cortex GSE35977	50 HC, 51 SZ	45.5 ± 8.99	42.65 ± 9.87	0.132	35/15	37/14	0.828	27.3 ± 11.82	31.39 ± 15.53	0.124	6.51 ± 0.31	6.37 ± 0.29	0.018*	-	-	-
Superior temporal cortex GSE21935	19 HC, 23 SZ	67.68 ± 22.24	72.17 ± 16.94	0.742	10/9	13/10	1	9.12 ± 4.33	7.13 ± 5.75	0.023*	6.49 ± 0.32	6.16 ± 0.17	0.001*	-	-	-
DLPFC -BA46 GSE53987	19 HC, 15 SZ	48.05 ± 10.65	46 ± 8.64	0.549	10/9	7/8	1	19.53 ± 5.09	18.91 ± 6.69	0.763	6.59 ± 0.22	6.53 ± 0.39	0.542	7.85 ± 0.62	7.65 ± 0.68	0.376
Frontal cortex GSE62191	30 HC, 29 SZ	43.5 ± 8.57	42.34 ± 8.95	NS^b^	23/7	23/6	1	29.97 ± 12.71	31.40 ± 16.93	NS^b^	6.61 ± 0.28	6.45 ± 0.25	0.01*^b^	-	-	-

*HC* healthy controls, *SZ* individuals with schizophrenia, *SD* standard deviation, *M/F* male/female, *RIN* RNA Integrity Number, *PFC* prefrontal cortex, *DLPFC* dorsolateral prefrontal cortex, *NA* not available, *NS* not significant.

*p < 0.05.

^a^Unpaired *t*-test and Fisher exact tests were conducted to assess group differences for continuous and discrete variables, respectively.

^b^As reported in ref. [101].

**Table 2:** Demographic and quality characteristics of the peripheral tissues.

Brain region	Subjects (n)	Age (mean ± SD)			Sex (M/F)		
		HC	SZ	*p*-value^1^	HC	SZ	*p*-value^1^
Blood (whole blood) GSE38484	96 HC, 106 SZ	39.31 ± 14.19	39.58 ± 10.74	0.538	42/54	76/30	0.001*
Blood (PBMCs) GSE27383	29 HC, 43 SZ	23.90 ± 4.08	23.02 ± 4.03	0.378	29/0	43/0	1
Skin fibroblasts GSE62333	20 HC, 20 SZ	48.4 ± 12.2	44.6 ± 12.67	0.340	9/11	10/10	1

*HC* healthy controls, *SZ* individuals with schizophrenia, *SD* standard deviation, *M/F* male/female, *PBMC* peripheral blood mononuclear cell.

*p < 0.05.

^a^Unpaired *t* test and Fisher exact tests were conducted to assess group differences for continuous and discrete variables, respectively.

### Ethical statement

All the data used in this project were acquired in previous studies, all of which conformed to ethical standards [26–33].

### Statistical analyses

JASP software (Version 0.11.1) was used to perform statistical analyses on gene expression data downloaded with shinyGEO. Gene expression values are already Log2-transformed to stabilize the variance and descriptive statistics of Log2 values in brain and peripheral tissues are provided in Supplementary Table 1. The fold change expression (FC) was computed as the difference between the average expression of HCs and the average expression of individuals with SZ.

Normal distribution and homogeneity of variances were tested using Shapiro-Wilk and Levene’s tests, respectively. For differential expression, exact *p*-values were calculated using unpaired two-tailed *t* tests. In case of violation of the equal variance assumption, exact *p*-values were calculated using Welch tests. When a deviation from normality was detected, exact *p*-values were calculated using Mann–Whitney *U* tests. The resulting *p*-values were adjusted with the Benjamini and Hochberg’s approach to control the false discovery rate. Gene expression comparisons were considered to be statistically significant for adjusted *p*-values < 0.05.

For genes that were significantly differentially expressed in individuals with SZ as compared with HCs, ANCOVA was used to evaluate the impact of potential confounding variables on candidate genes’ expression. Each candidate gene expression was separately entered as a dependent variable, the group (individuals with SZ or HCs) was used as a fixed factor, while demographic and quality characteristics, when available, were used as covariates (i.e., age, sex, RIN, brain pH, and postmortem interval).

### Bayesian analyses

Additional Bayesian analyses were performed. Particularly, Bayesian two-sample *t* tests were conducted in JASP for genes that were significantly differentially expressed in individuals with SZ as compared with HCs in frequentist analyses. These analyses allowed to quantify the evidence in favor of the alternative hypothesis (H_1_, hypothesizing the presence of a group difference) as well as the null hypothesis (H_0_, hypothesizing the absence of a group difference) through inspection of the Bayes Factors (BFs). See Supplementary Methods for more details on Bayesian analyses.

## Results

### Microglia genes

The present study investigated a pre-registered list of genes that are part of a core human microglia signatures [21, 22]. This list comprises the following 10 genes: *AIF1*, *CD68*, *CSF1R*, *CX3CR1*, *IRF8*, *ITGAX*, *NCF4*, *OLR1*, *TLR2, TMEM119*. Besides TMEM119 [34], all the included genes are also expressed in peripheral macrophages. See Fig. 1 and Supplementary Methods for details on selection of candidate genes.

### Included datasets

The systematic search in the GEO database, which identified records published until July 6th, 2022, yielded a total of 88 datasets (see Supplementary Fig. 1 for the flow chart). After applying inclusion criteria, the number of datasets was filtered to a total of 12 eligible datasets.

Eventually, 9 GEO datasets were included in the present study (see Supplementary Fig. 1 and Supplementary Methods for details on the selection procedure and exclusions). The included GEO datasets consist of 764 samples obtained from 266 individuals with SZ and 237 HCs. They provided data from 8 different brain regions relevant to SZ (cerebellum, associative striatum, hippocampus, anterior prefrontal cortex (BA10), dorsolateral prefrontal cortex (DLPFC, BA46), superior temporal cortex (BA22), and broad sections of the frontal cortex and parietal cortex) and 3 peripheral tissues (whole blood, peripheral blood mononuclear cells (PBMCs), and skin fibroblasts) (see Tables 1–2 for sample sizes, demographic and quality characteristics of included datasets). The original studies from which the included datasets were obtained are presented in Supplementary Table 2. It should be noted that included datasets were collected from different subjects.

### Differential expression analyses in postmortem brain tissues

**Fig. 2:**
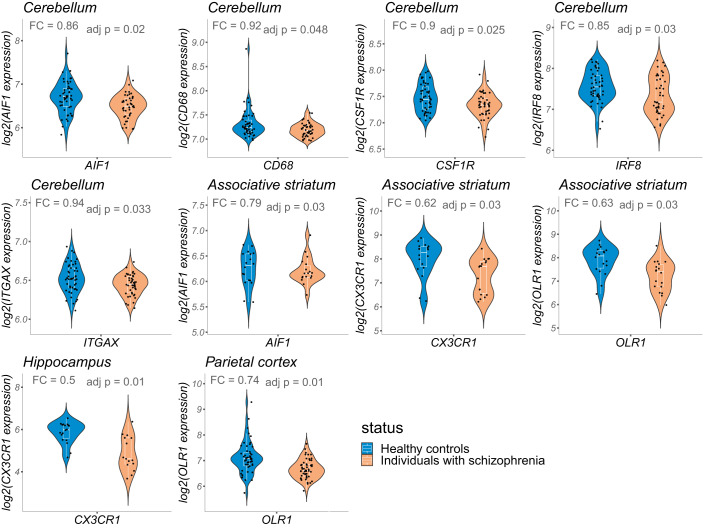
Differentially expressed genes in the postmortem brain samples of individuals with schizophrenia as compared with healthy controls. Shown are violin plots displaying gene expression distribution with overlaid box plots. For each gene, fold change (FC) represents the expression of the target gene in individuals with schizophrenia relative to that in healthy controls.

Distinct transcription patterns of microglia genes were identified at the brain level in individuals with SZ compared with HCs. Individuals with SZ exhibited a set of genes significantly underexpressed in the cerebellum (*AIF1*, *CD68*, *CSF1R*, *IRF8*, *ITGAX)*, the associative striatum (*AIF1*, *CX3CR1*, *OLR1)*, the hippocampus (*CX3CR1)* and parietal cortex (*OLR1)*. No alterations were found in DLPFC, anterior prefrontal cortex, frontal cortex, and superior temporal cortex of individuals with SZ as compared with HCs. Results are depicted in Fig. 2 and fold-change and adjusted p-values are detailed in Supplementary Table 3.

When taking into account several covariates (e.g., age, sex, RIN, brain pH, postmortem interval) with ANCOVA analyses, all the findings remained, except for the underexpression of *AIF1* in the associative striatum (see Supplementary Table 4).

### Differential expression analyses in peripheral tissues

**Fig. 3:**
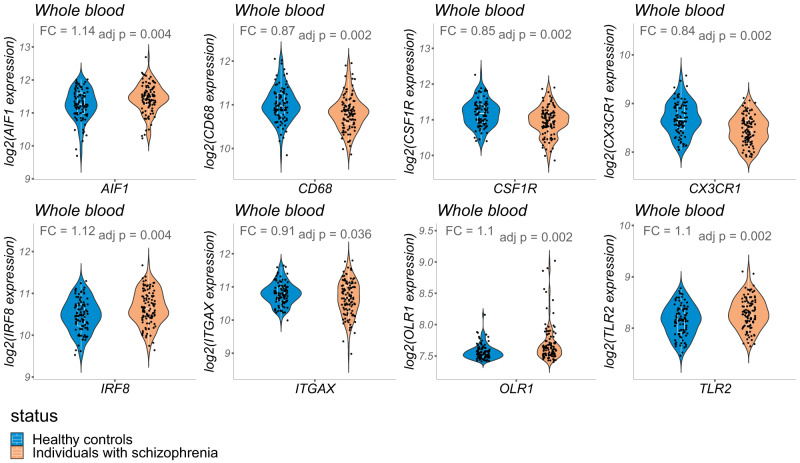
Differentially expressed genes in the peripheral blood samples of individuals with schizophrenia as compared with healthy controls. Shown are violin plots displaying gene expression distribution with overlaid box plots. For each gene, fold change (FC) represents the expression of the target gene in individuals with schizophrenia relative to that in healthy controls.

In the whole blood, we found a decreased expression of *CD68*, *CSF1R*, *CX3CR1*, and *ITGAX* in individuals with SZ compared with HCs. Also, a significant overexpression of *AIF1*, *IRF8*, *OLR1*, and *TLR2* was observed in the whole blood. No significant differences in expression of microglia genes were observed in the PBMCs and skin fibroblasts. Results are depicted in Fig. 3 and fold-change and adjusted *p*-values are detailed in Supplementary Table 5.

When taking into account several covariates (e.g., age and sex) with ANCOVA analyses, all the findings remained, except for the *ITGAX* underexpression in the whole blood (see Supplementary Table 6).

### Bayesian analyses

In postmortem brain tissues, using Bayesian analyses led to the same conclusions as frequentists statistics regarding the genes downregulated in SZ, with the exception of *AIF1* underexpression in the associative striatum of individuals with SZ compared with HCs (which was statistically significant in frequentist analysis whereas it was not possible to conclude in favor of the null or alternative hypotheses in Bayesian analyses). Notably, Bayesian analyses concluded with decisive evidence (BF_10_ > 100) for the downregulation of *CX3CR1* in the hippocampus and of *OLR1* in the parietal cortex of individuals with SZ compared with HCs (see Supplementary Table 4).

In peripheral tissues, Bayesian and frequentist analyses yielded the same conclusions, with the exception of *ITGAX* underexpression in the whole blood of individuals with SZ compared with HCs for which Bayesian analyses did not allow to conclude in favor of the null or alternative hypotheses. Noteworthily, Bayesian analyses concluded with decisive evidence (BF_10_ > 100) for the underexpression of *CX3CR1 and CSF1R* and overexpression of *OLR1* in the whole blood of individuals with SZ compared with HCs (see Supplementary Table 6).

## Discussion

In the present study we explored the expression of a pre-registered list of 10 genes from a core human signature of microglia in 8 different brain regions and in 3 peripheral tissues of individuals with SZ as compared with HCs. We observed altered expression patterns of the microglia-specific genes both in the brain and whole blood of individuals with SZ. The identified patterns were distinct between the different tissues. The present study allowed us to replicate and refine previous knowledge regarding SZ-associated microglial gene expression alterations as well as to expand such alteration to other brain regions and peripheral tissues.

### Transcriptional alterations of microglia genes in brain tissues

First, we report altered transcriptional patterns of microglia genes in the cerebellum, associative striatum, hippocampus, and parietal cortex of individuals with SZ compared with HCs. Notably, in these 4 latter brain regions, all the identified transcriptional alterations were underexpressions. This is consistent with a recent meta-analysis reporting underexpression of microglia genes in the brain of individuals with SZ [15]. While meta-analytic results from Snijders et al. [15] were obtained by pooling expression data collected from 4 different brain regions (frontal, temporal, cingulate cortices and hippocampus), we analyzed separately postmortem datasets from different brain regions, allowing for more precise localization of effects. Thus, our findings expand the previously described underexpression of microglia genes to 3 additional brain regions relevant to SZ pathogenesis (namely the cerebellum, associative striatum and parietal cortex).

Among the explored brain regions, the cerebellum was characterized by the greatest number of underexpressed microglia genes (*AIF1, CD68, CSF1R*, *IRF8*, and *ITGAX*). Noteworthily, in the healthy brain cerebellar microglia is characterized by a unique profile [35]. These results are consistent with the growing body of literature involving the cerebellum in SZ pathogenesis [36–38]. Indeed, beside its role in motor coordination and balance, the cerebellum is also implied in cognitive and emotional functions [39, 40], and cerebellar dysfunctions have been linked to SZ symptoms such as hallucinations [41].

To our knowledge, it is the first time that transcriptional alterations of microglia-specific genes are reported in the associative striatum and hippocampus of individuals with SZ, although studies using animal models of SZ reported microglial alterations in these latter regions [42–45]. Remarkably, previous research suggested that striatal and hippocampal SZ-associated alterations involve dendritic spine loss, synaptic alterations and/or impaired neurogenesis [46–49], potentially through microglial dysfunction [50]. Our present results lend support to this hypothesis in two brain regions where adult neurogenesis events occur [51, 52].

Similarly, this is the first study to report *OLR1* underexpression in the parietal cortex of individuals with SZ. This finding is consistent with the correlation between microglial activity and altered cortical gray matter volume previously found in the parietal cortex of individuals with SZ [53].

However, we did not replicate previous reports of decreased expression of microglia genes in the frontal and temporal cortex of individuals with SZ [15, 54, 55]. The discrepancy may be accounted for by the heterogeneity of the samples’ quality and demographic characteristics (such as differences in postmortem interval and sex distribution), sample size (our data were obtained from larger-sized samples as compared to the other studies) or techniques (we used microarray-derived data whereas these previous studies relied on quantitative real-time PCR techniques, it is therefore possible that alternative transcripts of the candidate genes were explored).

Overall, at the brain level, the consistency of the direction of the transcriptional alterations should be highlighted. Indeed, a recent systematic review investigating transcriptional alterations in SZ reported a high variability in the direction of the observed effect, with only few genes being consistently reported as over- or underexpressed across more than two or three studies [56]. Remarkably, in the present research, 3 genes (*AIF1*, *OLR1* and *CX3CR1*) showed the same direction of alteration in the analyzed brain samples. Most importantly, all the transcriptional alterations identified in 4 brain regions were underexpressions. In this light, our results suggest that brain underexpression of microglia genes may be considered as a candidate transcriptional signature for SZ. Furthermore, in individuals with SZ, the consistency of the direction of the transcriptional alterations contrasts with the heterogeneous results obtained using other microglia markers such as microglia activation, density, or morphology [13–16]. Our results thus strengthen the transcriptional analysis as a promising approach to explore SZ-associated microglia dysregulation.

Functionally, two alternative, non-exclusive hypotheses can be drawn regarding the identified underexpression patterns of microglia genes in the brain of individuals with SZ. As a first hypothesis, the observed transcriptional alterations may be related to the synaptic pruning impairment, which has been implicated in SZ pathogenesis [57]. Indeed, most of the candidate genes are involved in microglia key functions, such as “don’t eat me signals” expressed by neurons which maintains microglia in a homeostatic state (for *CX3CR1*) [58], or microglial migration, adhesion and motility (for *CX3CR1*, *IRF8* and *AIF1*) [59–61]. Hence, in the brain of individuals with SZ, *CX3CR1*, *IRF8* and/or *AIF1* underexpression may (i) either compromise the course of synaptic pruning by disrupting key microglial mechanisms [62] or (ii) reflect a regulatory attempt to counterbalance an excessive synaptic pruning in the brain of individuals with SZ [11, 63]. Alternatively, the identified transcriptional alterations may reflect a compensatory mechanism for neuroinflammation in the brain of individuals with SZ [64]. Consistently, animal models have involved several of our candidate genes (*CD68*, *CSF1R*, whose expression is necessary for microglia viability [65], *CX3CR1*, *AIF1*, *OLR1* and *ITGAX*) in inflammatory processes [66–72].

### Transcriptional alterations of microglia genes in peripheral tissues

At the peripheral level, no changes were found in PBMCs and skin fibroblasts, although they are considered as promising surrogate tissues for the study of SZ pathogenesis [73, 74]. Nevertheless, transcriptional alterations were identified in the whole blood of individuals with SZ, showing the co-existence of 4 underexpressed genes (*CD68*, *CSF1R*, *CX3CR1*, *ITGAX*) and 4 overexpressed ones (*AIF1*, *IRF8*, *OLR1*, *TLR2*). Although both whole blood and PBMCs are derived from blood, PBMCs remain a subset of all blood cells. Thus, variations in gene expression are expected between those two compartments since they exhibit distinct specific expression profiles [75]. Furthermore, PBMCs and whole blood samples used in the present study were not derived from the same subjects. Therefore, the discrepant results may be attributed to differences in their collection, storage, and extraction methods, as well as in heterogeneity of demographic characteristics of the included datasets [76].

In individuals with SZ, while all the candidate genes differentially expressed at the brain level were also differentially expressed in the whole blood, the peripheral transcriptional alterations only partially reflected the cerebral ones. Indeed, *AIF1*, *IRF8* and *OLR1* showed opposite directions of change between the two levels. Furthermore, *TLR2* was overexpressed in the whole blood contrasting with the absence of change at the brain level. Different factors may explain brain and whole blood discrepancy in gene expression changes. First, in this study, blood and brain samples were not derived from the same subjects. Second, since gene expression is not expected to be uniform across all the different brain regions [77, 78], blood and brain gene expression correlation may differ depending on the brain region. Third, SZ pathogenesis may not be restricted to brain tissue as SZ is now considered as a “whole-body” disorder [79], with distinct alterations at the brain and peripheral levels.

Remarkably, besides *TMEM119* [34], all the differentially expressed genes in the whole blood of individuals with SZ are expressed by peripheral macrophages and/or involved in the production, differentiation, activation, function and survival of peripheral macrophages [80–87]. Thus, our whole blood results support a SZ-associated dysregulation of peripheral macrophages, which is consistent with a previous report [88] and with accumulating evidence of an immune dysregulation involvement in SZ pathogenesis [89]. In individuals with SZ, the coexistence of a brain microglia alteration together with a whole blood dysregulation of peripheral macrophages is reported here for the first time, although some previous studies paved the way to the present finding. Indeed, at the brain level, North et al. [88] reported dual microglia-perivascular macrophages alterations in the brain of individuals with SZ. At the peripheral level, using induced microglia-like cells from peripheral macrophages, Ormel et al. [90] suggested altered developmental pathways of microglia in individuals with SZ. Overall, while our brain-peripheral results support the model of SZ as a whole-body disorder [79], more research is needed to determine whether the whole blood transcriptional alterations identified only reflect a SZ-associated dysregulation of peripheral macrophages or can be considered as a peripheral transcriptional signature of dual brain microglia-peripheral macrophages alterations in individuals with SZ.

### Limitations

Interpretation of the present results might be affected by limiting factors.

First, brain and peripheral tissues were obtained from a total of 9 different and modest-sized samples of patients with SZ and healthy controls. These modest sample sizes inherently limit statistical power, leading to an increased risk of generating spurious findings. Although measures were taken to mitigate this limitation, such as focusing on a specific list of candidate genes and pre-registering the analysis plan, the constraint imposed by the small sample sizes remains a crucial consideration. Additionally, the unavailability of independent validation datasets within the GEO database prevented us from replicating our results. Future studies with larger sample sizes are thus warranted to corroborate and expand the current findings, thereby providing more robust insights into the gene expression patterns of microglia associated with SZ. Second, it should be highlighted that SZ is a highly heterogeneous disorder. Notably, recent research suggests that heightened inflammation may be characteristic of a specific subgroup within the SZ population [88, 91–93]. Interestingly, a recent study investigating the gene expression of four microglia markers found that patients with SZ who demonstrated a high inflammatory profile showed a downregulation of CD68, in comparison to both patients with low inflammation and healthy controls [91]. However, due to the unavailability of clinical information and biomarker data within the GEO database, we were unable to perform an inflammatory profiling analysis to characterize these subgroups in our study. Therefore, future investigations should address the heterogeneity of SZ by exploring potential differential gene expression in these distinct subgroups.

Third, due the limited availability of demographic information provided with GEO datasets, we could not investigate the influence of some potential confounding variables. For instance, we could not explore the effect of smoking or of suicide as a cause of death although such factors have been shown to influence gene expression [94, 95] and enhance proinflammatory processes [96, 97]. Similarly, the effect of antipsychotic treatment could not be evaluated, while it has been suggested that it may inhibit microglia activation [98].

Fourth, all the included datasets were acquired through microarray technology. Our results are thus subject to the methodological limitations that lie within the use of microarrays: (i) background hybridization limit gene expression measurement, especially for low abundance transcripts, (ii) probe performance may have led to specificity issues (through cross-hybridization and non-specific hybridization), (iii) only genes for which probes are designed can be explored.

Finally, brain expression data were obtained from postmortem cerebral samples composed of heterogeneous cell populations instead of isolating pure microglia. Despite this limitation, it is noteworthy that all the candidate genes were part of a core transcriptional signature of human microglia [21], as well as part of a microglia signature highly expressed in bulk brain tissues [22]. Nonetheless it is essential to conduct further investigations using high-throughput sequencing techniques such as single-cell RNA-seq technology [99], which can provide insights into the heterogeneity and diversity of microglia populations.

### Conclusion

Using 9 gene expression datasets, we report evidence of a widespread transcriptional alteration of genes from a core human microglia signature both in brain tissues and whole blood of individuals with SZ compared with HCs. In brain tissues, we expand and refine previous knowledge regarding underexpression of microglia genes in the cerebellum, associative striatum, hippocampus, and parietal cortex. Such alterations may be considered as a candidate transcriptional signature for SZ disorders. At the peripheral level, we identified differential expression of the candidate genes in the whole blood of individuals with SZ. The dual brain-whole blood transcriptional alterations of microglia/macrophage genes identified in individuals with SZ support the model of SZ as a whole-body disorder and lend weight to the use of blood samples as a potential source of biological peripheral biomarkers. Subsequent investigations should thus focus on corroborating these findings within extensive population cohorts to enhance the robustness and generalizability of these conclusions.

### Supplementary information

Supplementary Information

## Data Availability

The data that support the findings of this study are openly available in Gene Expression Omnibus (NCBI) at https://www.ncbi.nlm.nih.gov/geo/, reference numbers GEO: GSE53987, GSE17612, GSE35977, GSE35974, GSE62191, GSE21935, GSE27383, GSE62333, GSE38484.
