# Supplementary material for: Molecular mapping of a core transcriptional signature of microglia-specific genes in schizophrenia
Source: Transl Psychiatry. 2023 Dec 13;13:386. doi: 10.1038/s41398-023-02677-y (PMC10719376; doi:10.1038/s41398-023-02677-y)
Supplement: Supplementary file 1 — Supplementary Information [file 41398_2023_2677_MOESM1_ESM.pdf]

SUPPLEMENTARY INFORMATION

Molecular mapping of a core transcriptional signature of microglia-specific genes in schizophrenia

Fiorito *et al.*

Contents:

Supplementary Methods 2

Microglia genes 2

Inclusion of datasets 3

Datasets included 4

Bayesian analyses 5

Supplementary Figure 1. Flow Chart outlining the selection procedure of GEO datasets 6

Supplementary Table 1. Descriptive Statistics of Log2 (gene expression) values in brain and peripheral tissues 7

Supplementary Table 2. Description of the original studies from which the datasets were obtained 13

Supplementary Table 3. Genes with altered expression in the postmortem brain samples of individuals with schizophrenia compared with healthy controls 20

Supplementary Table 4. Results of ANCOVA and Bayesian analyses in the postmortem brain samples of individuals with schizophrenia compared with healthy controls 21

Supplementary Table 5. Genes with altered expression in the peripheral tissue samples of individuals with schizophrenia compared with healthy controls 22

Supplementary Table 6. Results of ANCOVA and Bayesian analyses in the peripheral tissue samples of individuals with schizophrenia compared with healthy controls 23

References 24

Supplementary Methods

Microglia genes

The present study exclusively included genes that are part of a core transcriptional signature of human microglia [1]. This signature was established by Patir and colleagues through the identification of co-expressed genes associated with microglia, ensuring their presence in at least three out of nine distinct human datasets of microglia.

Among these genes, we only included microglia genes previously shown to be transcriptionally altered in SZ. We identified these genes according to three criteria.

Firstly, we selected the following genes whose expression was found to be altered in at least one study included in the meta-analysis conducted by Snijders et al. [2] (which explored the expression of 8 genes in total): *AIF1* (Hedges' g = -1.324, p-value = 0.003), *CD68* (Hedges' g = -0.845, p-value = 0.028), *CSF1R* (Hedges' g = -0.874, p-value = 0.023), *HLA-DRB4* (Hedges' g = -1.403, p-value = 0.014).

Secondly, we selected the following genes that were transcriptionnaly altered both in a postmortem study exploring 16 microglia genes from Snijders et al. [2] (*CSF1R* (Log_2FC = -3.306, adj p-value = <0.05), *IRF8* (Log_2FC = -2.945, adj p-value = <0.05), *ITGAX* (Log_2FC = -2.535, adj p-value = <0.05), *OLR1* (Log_2FC = -5.500, adj p-value = <0.05), *TMEM119* (Log_2FC = -6.532, adj p-value = <0.05)) and in the largest transcriptomic study in schizophrenia investigating the expression of 25774 genes, from Gandal et al. [3] (*CSF1R* (Log_2FC = -0.176, adj p-value = <0.05), *IRF8* (Log_2FC = -0.229, adj p-value = <0.05), *ITGAX* (Log_2FC = -0.305, adj p-value = <0.05), *OLR1* (Log_2FC = -0.215, adj p-value = <0.05), *TMEM119* (Log_2FC = -0.237, adj p-value = <0.05)): *CSF1R*, *IRF8*, *ITGAX*, *OLR1*, *TMEM119*.

Thirdly, we selected the following genes that consistently exhibited differential expression in individuals with schizophrenia compared with healthy controls in a meta-

analysis conducted by Bergon and collaborators [4] which explored the expression of 8655 genes in postmortem brain tissues (*CX3CR1* (FC = -1.24, adj p-value = < 0.001), *NCF4* (FC = 1.06, adj p-value = 0.0005), *TLR2* (FC = 1.12, adj p-value = 0.00013), *TSPO* (FC = 1.10, adj p-value = 0.0006)), as well as 16661 genes in peripheral tissues (*CX3CR1* (FC = -1.19, adj p-value = 0.0019), *NCF4* (FC = 1.13, adj p-value = 0.017), *TLR2* (FC = 1.16, adj p-value = 0.0007), *TSPO* (FC = 1.12, adj p-value = 0.012)): *CX3CR1*, *NCF4*, *TLR2*.

HLA-DRB4 was not included due to the lack of specificity of the microarray probes, and TSPO was omitted as its relevance as a microglia marker has been questioned [5] with evidence suggesting a closer association with astrocytes [6].

Eventually, it should be noted that the genes included in this study exceeded the minimum requirement of being present in three datasets, as established by Patir et al. when defining the core transcriptional signature of human microglia [1]. Indeed, our candidate genes were present in a larger number of datasets, specifically in at least six out of the nine co-expression derived datasets, thus reinforcing their strong association with microglia. Additionally, it is noteworthy that all the genes included in this study were also identified in a recently published list of microglia signature genes that are highly expressed in bulk brain tissues [7].

Inclusion of datasets

Since our primary aim was to map transcriptional alterations of microglia genes in brain and peripheral tissues samples, one dataset per brain region (or peripheral tissue) was selected. When more than one dataset from the same brain region (or peripheral tissue) was available, we selected the one that would maximize the following 2 criteria in this particular order of relevance: 1) the dataset is capable to explore the largest

number of genes from our list of candidate genes; 2) the dataset has the largest number of subjects. Based on these criteria, 2 datasets (GSE21138 from Narayen et al., 2008 [8]; GSE38481 from de Jong et al., 2012 [9]) from the list of 12 eligible datasets were not included in the main analyses.

Datasets included

It should be noted that the datasets included in this study are slightly different from those that were pre-registered on AsPredicted.org (#67610, https://aspredicted.org/285rn.pdf). This is due to the following reasons: 1) we initially planned to include datasets using three types of Affymetrix arrays (HG–U133_Plus_2, Human Gene 1.0 ST or Human Gene 1.1 ST) which could technically interrogate our list of candidate genes. However, this criterion precluded the inclusion of other array platforms capable of exploring these candidate genes (such as Agilent or Illumina arrays). Therefore, this criterion was removed, and only custom-designed microarrays were excluded. Consequently, an additional non pre-registered dataset was included in this study (GSE62191 from de Baumont et al., 2015 [10]); 2) we excluded one pre-registered dataset (GSE93987 from Arion et al., 2015 [11] since it was captured through a laser microdissection of pyramidal cells, and thus did not contain microglia cells violating our inclusion criteria; 3) we also excluded one pre-registered dataset (GSE73129 from Horiuchi et al., 2016 [12]) due to lack of probe accuracy of at least half of the candidate genes (i.e., 5 genes present a lack of variability in gene expression values for all participants, probably reflecting transcriptional noise).

Following this selection, we report results from 9 different datasets. Finally, it should be noted that for two included datasets, we could not reliably measure the expression

of a few genes (*CD68* and *ITGAX* in the superior temporal cortex; *NCF4* and *TMEM119* in the frontal cortex) due to a lack of probe accuracy for those genes. The presently reported non-significant differences between individuals with schizophrenia and healthy controls for those latter genes in the relevant datasets should therefore be considered with caution.

Bayesian analyses

In additional Bayesian analyses, we quantify evidence in favor of the null (H_0) and alternative (H_1) hypotheses using the Bayes Factor (BF). Indeed, BFs are the ratio of the likelihood of the data under the alternative hypothesis and under the null hypothesis. BF_{10} quantifies the evidence in favor of H_1 compared with H_0, while BF_{01} (= $1/BF_{10}$) quantifies the evidence in favor of H_0 compared with H_1. Conventionally a BF_{10} (or BF_{01}) that exceeds the threshold of 3 represents moderate evidence in favor of H_1 (or H_0), while when it exceeds the threshold of 100 the evidence can be considered as decisive. Finally, it is not possible to conclude regarding the presence or absence of group differences when BF_{10} (or BF_{01}) is between 1 and 3 (i.e., anecdotal evidence) [13].

Supplementary Figure 1. Flow Chart outlining the selection procedure of GEO datasets

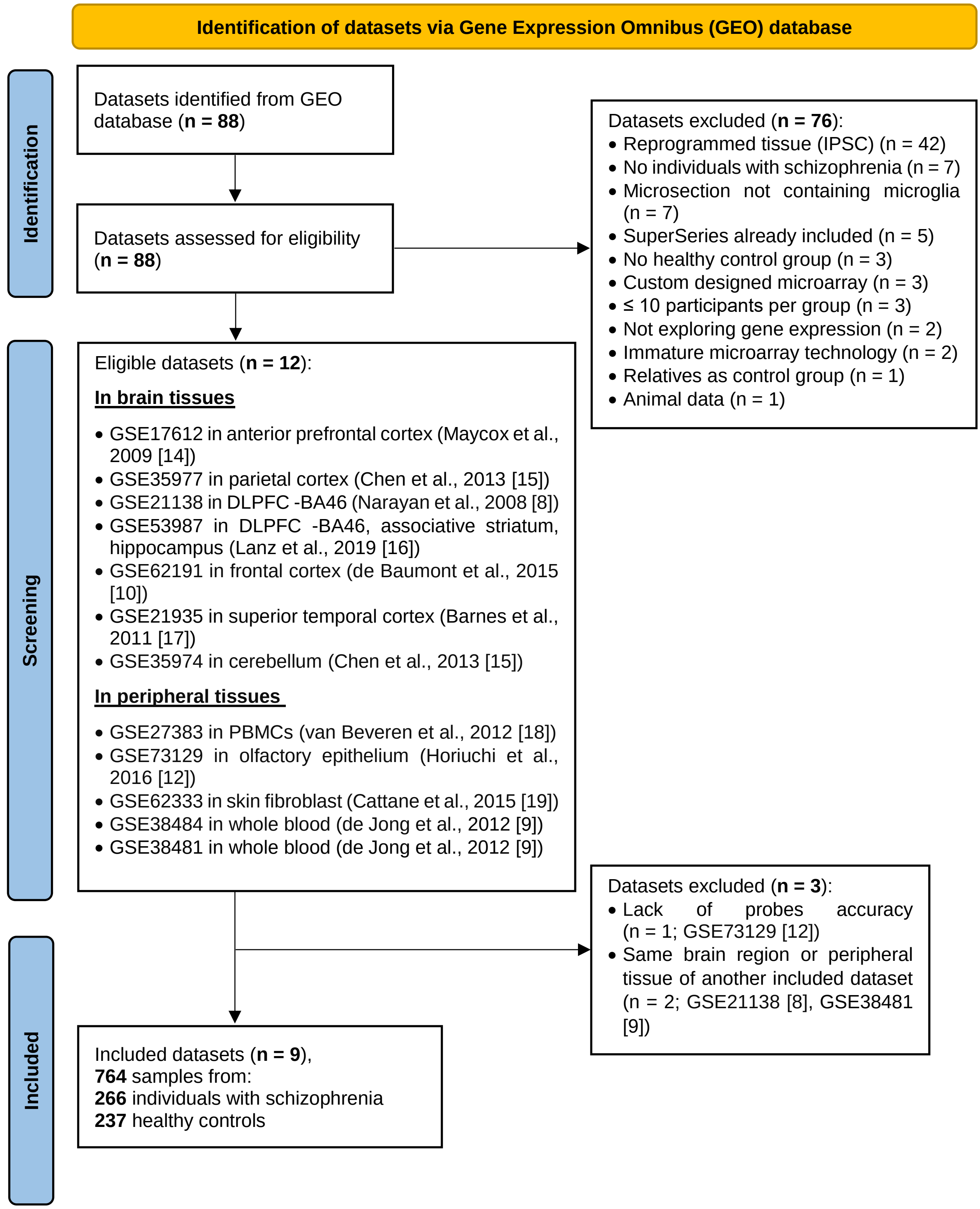

Abbreviations: GEO, Gene Expression Omnibus; IPSC, induced pluripotent stem cells; DLPFC, dorsolateral prefrontal cortex; BA, Brodmann area; PBMCs, peripheral blood mononuclear cells

Supplementary Table 1. Descriptive Statistics of Log2 (gene expression) values in brain and peripheral tissues

Anterior prefrontal cortex (GSE17612)																				
	Log2(AIF1)		Log2(CD68)		Log2(CSF1R)		Log2(CX3CR1)		Log2(IRF8)		Log2(ITGAX)		Log2(NCF4)		Log2(OLR1)		Log2(TLR2)		Log2(TMEM119)	
	HC	SZ	HC	SZ	HC	SZ	HC	SZ	HC	SZ	HC	SZ	HC	SZ	HC	SZ	HC	SZ	HC	SZ
Valid	23	28	23	28	23	28	23	28	23	28	23	28	23	28	23	28	23	28	23	28
Missing	0	0	0	0	0	0	0	0	0	0	0	0	0	0	0	0	0	0	0	0
Mean	4.21	4.74	3.78	3.68	7.99	8.22	8.06	8.27	6.47	6.78	5.47	5.65	5.91	5.88	6.47	6.57	5.30	6.01	5.79	6.09
SD	0.58	1.09	0.90	0.89	0.58	0.51	0.89	0.86	0.51	0.48	0.88	0.58	0.59	0.62	0.37	0.57	1.42	1.32	0.56	0.44
Normality	No	No	Yes	Yes	Yes	Yes	Yes	Yes	Yes	Yes	Yes	Yes	Yes	No	Yes	No	Yes	Yes	Yes	Yes
Homoscedasticity	No		Yes		Yes		Yes		Yes		No		Yes		Yes		Yes		Yes	
Max	6.24	7.06	5.74	6.04	9.11	9.10	9.23	9.79	7.41	7.58	6.83	6.70	6.85	6.78	7.27	9.01	7.47	8.00	6.85	7.05
Min	3.68	3.31	2.48	2.40	6.72	7.36	6.18	5.99	5.68	5.80	3.65	4.22	4.14	4.01	5.46	5.95	1.80	2.82	4.62	4.92

Parietal cortex (GSE35977)																				
	Log2(AIF1)		Log2(CD68)		Log2(CSF1R)		Log2(CX3CR1)		Log2(IRF8)		Log2(ITGAX)		Log2(NCF4)		Log2(OLR1)		Log2(TLR2)		Log2(TMEM119)	
	HC	SZ	HC	SZ	HC	SZ	HC	SZ	HC	SZ	HC	SZ	HC	SZ	HC	SZ	HC	SZ	HC	SZ
Valid	50	51	50	51	50	51	50	51	50	51	50	51	50	51	50	51	50	51	50	51
Missing	0	0	0	0	0	0	0	0	0	0	0	0	0	0	0	0	0	0	0	0
Mean	6.60	6.44	7.15	7.08	7.69	7.55	7.32	7.04	7.14	6.99	6.46	6.34	5.61	5.58	7.11	6.67	5.97	6.04	6.55	6.47
SD	0.49	0.43	0.48	0.40	0.49	0.47	0.85	0.72	0.50	0.45	0.30	0.24	0.20	0.23	0.63	0.38	0.57	0.47	0.22	0.17
Normality	No	Yes	No	No	No	Yes	Yes	Yes	No	No	No	Yes	Yes	Yes	No	Yes	No	No	Yes	Yes
Homoscedasticity	Yes		Yes		Yes		Yes		Yes		Yes		Yes		No		Yes		Yes	
Max	8.80	7.59	9.14	8.38	9.59	8.54	9.14	8.45	9.13	8.53	7.81	6.92	6.26	6.27	9.28	7.66	9.18	7.87	7.09	6.88
Min	5.89	5.32	6.69	6.47	6.89	6.42	5.69	5.61	6.19	6.17	6.04	5.87	5.24	5.17	5.74	5.82	5.40	5.54	6.14	6.01

Superior Temporal cortex (GSE21935)																				
	Log2(AIF1)		Log2(CD68)		Log2(CSF1R)		Log2(CX3CR1)		Log2(IRF8)		Log2(ITGAX)		Log2(NCF4)		Log2(OLR1)		Log2(TLR2)		Log2(TMEM119)	
	HC	SZ	HC	SZ	HC	SZ	HC	SZ	HC	SZ	HC	SZ	HC	SZ	HC	SZ	HC	SZ	HC	SZ
Valid	13	18	3	2	19	23	19	23	19	23	5	9	17	23	19	23	19	22	19	23
Missing	6	5	16	21	0	0	0	0	0	0	14	14	2	0	0	0	0	1	0	0
Mean	3.78	3.31	1.57	1.98	6.74	7.16	7.27	7.54	4.71	5.63	3.35	2.78	4.19	4.61	5.42	5.63	4.28	5.13	3.95	4.28
SD	1.25	1.15	2.05	0.60	0.76	0.85	0.79	1.13	1.11	0.96	1.31	2.10	0.67	0.59	0.53	0.68	1.15	0.98	1.21	1.38
Normality	Yes	Yes	NA	NA	Yes	Yes	Yes	Yes	Yes	Yes	NA	NA	Yes	Yes	Yes	No	Yes	Yes	Yes	No
Homoscedasticity	Yes		NA		Yes		Yes		Yes		NA		Yes		Yes		Yes		Yes	
Max	5.27	5.09	3.78	2.40	8.73	8.53	9.16	9.32	7.07	6.74	4.43	4.71	5.16	5.64	6.13	7.76	6.05	6.40	6.32	5.99
Min	0.97	1.07	-0.27	1.55	5.34	5.27	5.66	5.58	2.40	3.49	1.59	-1.15	2.98	3.72	4.19	4.62	1.11	2.91	1.18	0.81
**Associative striatum (GSE53987)**																				
	Log2(AIF1)		Log2(CD68)		Log2(CSF1R)		Log2(CX3CR1)		Log2(IRF8)		Log2(ITGAX)		Log2(NCF4)		Log2(OLR1)		Log2(TLR2)		Log2(TMEM119)	
	HC	SZ	HC	SZ	HC	SZ	HC	SZ	HC	SZ	HC	SZ	HC	SZ	HC	SZ	HC	SZ	HC	SZ
Valid	18	18	18	18	18	18	18	18	18	18	18	18	18	18	18	18	18	18	18	18
Missing	0	0	0	0	0	0	0	0	0	0	0	0	0	0	0	0	0	0	0	0
Mean	6.21	6.25	7.37	7.45	9.06	8.89	8.01	7.31	7.61	7.73	7.24	7.16	6.98	7.06	7.89	7.22	6.89	7.01	6.71	6.50
SD	0.26	0.34	0.15	0.17	0.38	0.54	0.75	0.73	0.29	0.38	0.17	0.20	0.09	0.29	0.61	0.65	0.29	0.54	0.29	0.41
Normality	Yes	Yes	Yes	No	Yes	Yes	No	No	Yes	No	Yes	Yes	Yes	No	Yes	Yes	No	No	Yes	Yes
Homoscedasticity	Yes		Yes		Yes		Yes		Yes		Yes		No		Yes		Yes		Yes	
Max	6.91	6.72	7.64	7.94	9.63	10.28	8.87	8.43	8.16	8.84	7.58	7.54	7.15	7.97	8.73	8.51	7.89	9.06	7.22	7.53
Min	5.73	5.59	7.11	7.26	8.37	7.94	6.24	6.21	7.20	7.03	6.90	6.69	6.79	6.62	6.45	5.98	6.55	6.67	6.34	5.80

Hippocampus (GSE53987)

	Log2(AIF1)		Log2(CD68)		Log2(CSF1R)		Log2(CX3CR1)		Log2(IRF8)		Log2(ITGAX)		Log2(NCF4)		Log2(OLR1)		Log2(TLR2)		Log2(TMEM119)	
	HC	SZ	HC	SZ	HC	SZ	HC	SZ	HC	SZ	HC	SZ	HC	SZ	HC	SZ	HC	SZ	HC	SZ
Valid	18	15	18	15	18	15	18	15	18	15	18	15	18	15	18	15	18	15	18	15
Missing	0	0	0	0	0	0	0	0	0	0	0	0	0	0	0	0	0	0	0	0
Mean	3.61	3.60	5.10	5.24	7.47	7.25	5.85	4.87	5.92	5.92	5.45	5.24	4.24	4.38	5.36	4.89	5.39	5.72	4.03	3.95
SD	0.24	0.20	0.23	0.18	0.42	0.85	0.50	0.818	0.30	0.60	0.25	0.36	0.20	0.48	0.45	0.97	0.39	1.06	0.21	0.53
Normality	Yes	Yes	Yes	Yes	Yes	Yes	No	Yes	Yes	Yes	No	Yes	Yes	No	Yes	No	Yes	No	Yes	Yes
Homoscedasticity	Yes		Yes		No		No		No		Yes		No		No		No		No	
Max	4.04	4.00	5.63	5.58	8.37	8.59	6.53	6.37	6.37	7.38	6.04	6.02	4.63	5.50	6.51	7.77	6.38	8.31	4.34	4.84
Min	3.21	3.26	4.70	5.01	6.79	6.08	4.68	3.68	5.32	5.07	5.17	4.68	3.75	3.81	4.78	4.04	4.91	4.90	3.66	3.19

DLPFC (GSE53987)

	Log2(AIF1)		Log2(CD68)		Log2(CSF1R)		Log2(CX3CR1)		Log2(IRF8)		Log2(ITGAX)		Log2(NCF4)		Log2(OLR1)		Log2(TLR2)		Log2(TMEM119)	
	HC	SZ	HC	SZ	HC	SZ	HC	SZ	HC	SZ	HC	SZ	HC	SZ	HC	SZ	HC	SZ	HC	SZ
Valid	19	15	19	15	19	15	19	15	19	15	19	15	19	15	19	15	19	15	19	15
Missing	0	0	0	0	0	0	0	0	0	0	0	0	0	0	0	0	0	0	0	0
Mean	3.53	3.46	4.93	5.00	7.25	7.12	5.53	5.05	5.89	5.76	4.68	4.58	4.54	4.57	5.76	5.52	5.00	5.40	3.88	3.93
SD	0.16	0.23	0.16	0.19	0.49	0.63	0.53	0.49	0.39	0.57	0.17	0.18	0.18	0.24	0.40	0.37	0.25	0.86	0.32	0.47
Normality	Yes	Yes	Yes	Yes	Yes	Yes	Yes	Yes	Yes	No	Yes	No	Yes	Yes	Yes	Yes	Yes	No	Yes	Yes
Homoscedasticity	Yes		Yes		Yes		Yes		Yes		Yes		Yes		Yes		No		Yes	
Max	3.87	3.90	5.22	5.27	8.08	8.53	6.24	5.74	6.64	7.55	4.94	4.81	4.94	5.23	6.52	6.24	5.64	7.50	4.51	5.05
Min	3.23	3.08	4.70	4.74	6.45	6.08	4.30	4.27	5.31	5.20	4.19	4.24	4.18	4.19	4.89	5.05	4.53	4.77	3.29	3.21

	Log2(AIF1)		Log2(CD68)		Log2(CSF1R)		Log2(CX3CR1)		Log2(IRF8)		Log2(ITGAX)		Log2(NCF4)		Log2(OLR1)		Log2(TLR2)		Log2(TMEM119)	
**Frontal cortex (GSE62191)**																				
	**HC**	**SZ**	**HC**	**SZ**	**HC**	**SZ**	**HC**	**SZ**	**HC**	**SZ**	**HC**	**SZ**	**HC**	**SZ**	**HC**	**SZ**	**HC**	**SZ**	**HC**	**SZ**
Valid	30	29	30	29	29	29	30	29	30	28	30	28	/	/	30	29	30	29	/	/
Missing	0	0	0	0	1	0	0	0	0	1	0	1	/	/	0	0	0	0	/	/
Mean	3.10	3.11	1.86	1.86	3.53	3.61	2.84	2.91	1.18	1.18	1.21	1.05	/	/	1.79	1.67	1.76	1.83	/	/
SD	0.35	0.24	0.34	0.26	0.37	0.30	0.51	0.39	0.33	0.20	0.30	0.25	/	/	0.32	0.22	0.47	0.37	/	/
Normality	Yes	Yes	Yes	Yes	Yes	No	Yes	No	No	Yes	Yes	No	/	/	Yes	Yes	No	Yes	/	/
Homoscedasticity	No		Yes		Yes		No		Yes		Yes		/	/	Yes		Yes		/	/
Max	3.84	3.58	2.60	2.62	4.19	4.08	3.77	3.58	1.98	1.51	1.82	1.49	/	/	2.69	2.01	3.05	2.68	/	/
Min	2.55	2.40	1.16	1.43	2.68	2.55	1.87	1.66	0.82	0.84	0.76	0.74	/	/	1.21	1.09	0.97	1.06	/	/
**Cerebellum (GSE35974)**																				
	**Log2(AIF1)**		**Log2(CD68)**		**Log2(CSF1R)**		**Log2(CX3CR1)**		**Log2(IRF8)**		**Log2(ITGAX)**		**Log2(NCF4)**		**Log2(OLR1)**		**Log2(TLR2)**		**Log2(TMEM119)**	
	**HC**	**SZ**	**HC**	**SZ**	**HC**	**SZ**	**HC**	**SZ**	**HC**	**SZ**	**HC**	**SZ**	**HC**	**SZ**	**HC**	**SZ**	**HC**	**SZ**	**HC**	**SZ**
Valid	50	44	50	44	50	44	50	44	50	44	50	44	50	44	50	44	50	44	50	44
Missing	0	0	0	0	0	0	0	0	0	0	0	0	0	0	0	0	0	0	0	0
Mean	6.70	6.48	7.32	7.19	7.48	7.33	6.93	6.81	7.57	7.33	6.53	6.44	6.11	6.11	5.67	5.51	5.99	5.93	7.00	6.98
SD	0.36	0.27	0.30	0.14	0.26	0.23	0.52	0.44	0.38	0.46	0.19	0.13	0.17	0.12	0.34	0.24	0.29	0.22	0.15	0.14
Normality	Yes	Yes	No	Yes	Yes	Yes	Yes	Yes	Yes	Yes	Yes	Yes	Yes	Yes	No	Yes	No	Yes	Yes	No
Homoscedasticity	Yes		No		Yes		Yes		No		Yes		No		Yes		Yes		Yes	
Max	7.71	7.08	8.87	7.54	7.98	7.92	8.01	7.69	8.22	8.19	6.93	6.74	6.52	6.39	6.58	6.01	7.39	6.46	7.38	7.19
Min	5.84	5.97	6.98	6.97	7.05	6.73	5.88	5.99	6.53	6.56	6.11	6.14	5.84	5.89	5.17	5.03	5.58	5.49	6.71	6.55

Blood -whole blood (GSE38484)																				
	Log2(AIF1)		Log2(CD68)		Log2(CSF1R)		Log2(CX3CR1)		Log2(IRF8)		Log2(ITGAX)		Log2(NCF4)		Log2(OLR1)		Log2(TLR2)		Log2(TMEM119)	
	HC	SZ	HC	SZ	HC	SZ	HC	SZ	HC	SZ	HC	SZ	HC	SZ	HC	SZ	HC	SZ	HC	SZ
Valid	96	106	96	106	96	106	96	106	96	106	96	106	96	106	96	106	96	106	96	106
Missing	0	0	0	0	0	0	0	0	0	0	0	0	0	0	0	0	0	0	0	0
Mean	11.25	11.44	11.01	10.82	11.18	10.95	8.69	8.45	10.48	10.65	10.80	10.66	8.96	8.94	7.57	7.70	8.15	8.29	7.50	7.47
SD	0.46	0.43	0.39	0.39	0.37	0.41	0.34	0.30	0.39	0.41	0.34	0.53	0.34	0.37	0.12	0.30	0.30	0.30	0.12	0.09
Normality	No	Yes	Yes	Yes	Yes	Yes	Yes	Yes	Yes	Yes	Yes	Yes	No	Yes	No	No	Yes	Yes	No	Yes
Homoscedasticity	Yes		Yes		Yes		Yes		Yes		No		Yes		No		Yes		Yes	
Max	12.02	12.69	12.05	11.95	12.26	11.90	9.57	9.11	11.30	11.67	11.65	11.80	9.92	9.81	8.16	9.02	8.78	9.10	8.09	7.75
Min	9.70	10.23	9.85	9.86	10.40	9.86	8.04	7.90	9.53	9.64	9.99	8.98	8.39	8.15	7.41	7.40	7.47	7.65	7.30	7.28
**Blood -PBMCs (GSE27383)**																				
	Log2(AIF1)		Log2(CD68)		Log2(CSF1R)		Log2(CX3CR1)		Log2(IRF8)		Log2(ITGAX)		Log2(NCF4)		Log2(OLR1)		Log2(TLR2)		Log2(TMEM119)	
	HC	SZ	HC	SZ	HC	SZ	HC	SZ	HC	SZ	HC	SZ	HC	SZ	HC	SZ	HC	SZ	HC	SZ
Valid	29	43	29	43	29	43	29	43	29	43	29	43	29	43	29	43	29	43	29	43
Missing	0	0	0	0	0	0	0	0	0	0	0	0	0	0	0	0	0	0	0	0
Mean	4.65	4.64	8.57	8.63	10.66	10.61	13.51	13.34	11.66	11.71	8.87	8.89	9.26	9.35	4.23	4.34	10.71	10.93	3.79	3.88
SD	0.11	0.15	0.31	0.44	0.29	0.34	0.29	0.26	0.22	0.18	0.22	0.28	0.34	0.35	0.32	0.57	0.36	0.45	0.13	0.15
Normality	Yes	No	Yes	Yes	Yes	Yes	Yes	No	Yes	Yes	Yes	Yes	Yes	Yes	No	No	Yes	No	Yes	Yes
Homoscedasticity	Yes		Yes		Yes		Yes		Yes		Yes		Yes		Yes		Yes		Yes	
Max	4.87	5.24	9.42	9.49	11.11	11.24	14.05	13.73	12.00	12.21	9.34	9.39	9.72	10.05	5.57	6.81	11.26	12.02	4.04	4.28
Min	4.39	4.36	8.08	7.17	10.18	9.45	12.94	12.38	11.21	11.30	8.52	8.03	8.57	8.54	3.91	3.84	9.83	9.72	3.53	3.60

Skin fibroblast (GSE62333)																				
	**Log2(AIF1)**		**Log2(CD68)**		**Log2(CSF1R)**		**Log2(CX3CR1)**		**Log2(IRF8)**		**Log2(ITGAX)**		**Log2(NCF4)**		**Log2(OLR1)**		**Log2(TLR2)**		**Log2(TMEM119)**	
	**HC**	**SZ**	**HC**	**SZ**	**HC**	**SZ**	**HC**	**SZ**	**HC**	**SZ**	**HC**	**SZ**	**HC**	**SZ**	**HC**	**SZ**	**HC**	**SZ**	**HC**	**SZ**
Valid	20	20	20	20	20	20	20	20	20	20	20	20	20	20	20	20	20	20	20	20
Missing	0	0	0	0	0	0	0	0	0	0	0	0	0	0	0	0	0	0	0	0
Mean	3.04	3.10	9.52	9.49	4.14	4.07	3.75	3.71	3.33	3.37	3.76	3.718	4.355	4.310	5.564	5.677	2.986	3.023	9.453	9.765
SD	0.10	0.16	0.72	0.51	0.23	0.18	0.22	0.35	0.30	0.30	0.177	0.123	0.156	0.192	1.009	1.011	0.093	0.128	0.473	0.468
Normality	Yes	Yes	No	Yes	No	Yes	No	No	Yes	Yes	Yes	Yes	Yes	Yes	Yes	Yes	Yes	Yes	Yes	Yes
Homoscedasticity	Yes		Yes		Yes		Yes		Yes		Yes		Yes		Yes		Yes		Yes	
Max	3.26	3.42	10.27	10.50	4.55	4.38	4.31	4.98	3.95	3.96	4.054	4.017	4.567	4.609	7.745	7.346	3.183	3.399	10.398	10.597
Min	2.85	2.89	7.70	8.71	3.81	3.63	3.43	3.40	2.94	2.87	3.365	3.535	3.973	3.910	3.806	3.877	2.826	2.783	8.272	8.804

Abbreviations: HC, healthy controls; SZ, individuals with schizophrenia; SD, Standard deviation; DLPFC, dorsolateral prefrontal cortex; PBMCs, Peripheral blood mononuclear cells

Supplementary Table 2. Description of the original studies from which the datasets were obtained

GEO Accession Number	Tissue	Region / Cell Type	Microarray	SZ	HC
GSE53987	Brain	DLPFC (BA46)	HG-U133_Plus_2	15	19
GSE53987	Brain	Hippocampus	HG-U133_Plus_2	15	18
GSE53987	Brain	Associative striatum	HG-U133_Plus_2	18	18

Description of the original study	
**Sample collection:**	University of Pittsburgh brain bank
**Consent / Ethics:**	Brain specimens were obtained during autopsies after consent for donation was obtained from the next-of-kin.<br>All procedures were approved by the University of Pittsburgh Committee for the Oversight of Research and Clinical Trials Involving the Dead and the Institutional Review Board for Biomedical Research
**SZ:**	Diagnoses were made by an independent committee of experienced research clinicians, using DSM-IV criteria and based on the results of structured interviews conducted with family members and review of medical records
**Healthy controls:**	The absence of psychiatric diagnoses was confirmed using an identical approach. The healthy controls were free of any neurological or psychiatric illness during their life course
**Original study:**	Lanz *et al*., Translational Psychiatry, 2019 (PMID: 31123247)
**Objective:**	To evaluate shared transcriptional alterations across connected brain regions in SZ, bipolar disorder (BD), major depressive disorder (MDD) individuals, or HC
**Methods:**	Genome-wide expression was obtained from postmortem DLPFC, hippocampus, and associative striatum from 19 well-matched tetrads of SZ, BD, MDD, or HC
**Main results:**	SZ showed a substantial burden of differentially expressed genes across all examined brain regions with the greatest effects in hippocampus, whereas BD and MDD showed less robust alterations. Pathway analysis of transcriptional profiles compared across diagnoses demonstrated commonly enriched pathways between all three disorders in hippocampus, significant overlap between SZ and BD in DLPFC, but no significant overlap of enriched pathways between disorders in striatum. SZ showed increased expression of transcripts associated with inflammation across all brain regions examined, which was not evident in BD or MDD individuals

GEO Accession Number	Tissue	Region / Cell Type	Microarray	SZ	HC	Description of the original study
GSE35977	Brain	Parietal cortex	HuGene-1_0-st	51	50	**Sample collection:** Stanley Medical Research Institute's Neuropathology Consortium and Array Collections<br>**Consent / Ethics:** Specimens were collected with informed consent from next-of-kin<br>**SZ:** Diagnoses were made by two senior psychiatrists, using DSM-IV criteria and based on medical records and, when necessary, telephone interviews with family members<br>**HC:** Diagnoses of unaffected controls were based on structured interviews by a senior psychiatrist with family member(s) to rule out Axis I diagnoses.<br>**Exclusion criteria:** Individuals over age 65 were excluded<br>**Original study:** Chen *et al*., Molecular Psychiatry, 2013 (PMID: 23147385)<br>**Objective:** To identify schizophrenia-associated gene-expression networks in the parietal cortex and cerebellum of SZ and HC. To test whether the gene modules perturbed in SZ were similarly perturbed in bipolar disorder<br>**Methods:** Genome-wide expression data was used to construct gene expression networks and identify gene co-expression modules within the networks. The modules were tested for association with SZ. Identified SZ-associated modules were tested for association with bipolar disorder<br>**Main results:** Two modules were differentially expressed in SZ versus HC. One, upregulated in cerebral cortex, was enriched with neuron differentiation and neuron development genes, as well as disease genome-wide association study genetic signals; the second, altered in cerebral cortex and cerebellum, was enriched with genes involved in neuron protection function. The findings were preserved in five expression datasets, including sets from three brain regions, from a different microarray platform, and from individuals with bipolar disorder
GSE35974	Brain	Cerebellum	HuGene-1_0-st	44	50	

GEO Accession Number	Tissue	Region / Cell Type	Microarray	SZ	HC	Description of the original study		
						**Sample collection:**	Tissue collection of the Charing Cross Hospital, Imperial College London, UK	
						**Consent / Ethics:**	All patients with the agreement of their nearest relative or authorized representative, have given written informed consent for use of tissue obtained post-mortem for research. The control group were tissue donors for research from the community. Procedures have been approved by the West London Mental Health Ethical Research Committee and complies with the conditions of the Research Governance Office of the Imperial College of Science, Technology and Medicine Clinical Research Office	
GSE17612	Brain	Anterior prefrontal cortex (BA10)	HG-U133_Plus_2	28	23	**SZ:**	All patients met DSM-III diagnostic criteria for chronic residual SZ with pronounced negative symptoms alongside attenuated positive symptoms and intellectual dysfunction. All patients had been treated with neuroleptic drugs except one patient who was neuroleptic naive at death	
						**HC:**	Mentally normal individuals from the community	
						**Exclusion criteria:**	Alzheimer's disease, Parkinson's disease or multiple sclerosis were excluded	
						**Original studies:**	Maycox *et al.*, Mol Psychiatry, 2009 (PMID: 19255580)	Barnes *et al.*, J Neurosci Res, 2011 (PMID: 21538462)
						**Objective:**	To identify differentially expressed genes in anterior prefrontal cortex (BA 10) from SZ and HC	Gene ontology pathway enrichment analysis in BA22 and BA10 from SZ and HC
GSE21935	Brain	Superior temporal cortex (BA22)	HG-U133_Plus_2	23	19	**Methods:**	Genome-wide expression in post-mortem brain tissue from anterior prefrontal cortex (BA 10) was compared between 28 SZ and 23 HC. Results were then compared to those from an independent prefrontal cortex dataset obtained from SZ and HC	Genome-wide expression was determined in the post-mortem BA22 region of 23 SZ and 19 HC and compared with genome-wide expression of BA10 from the same subjects. Gene ontology pathway enrichment analysis was carried out in each region
						**Main results:**	51 gene expression changes were common between the two SZ cohorts, and 49 showed the same direction of disease-associated regulation. Changes were observed in gene sets	In BA22 region, the highest enrichment was observed in processes mediating cell adhesion, synaptic contact, cytoskeletal remodeling, and apoptosis. In BA10 region, the strongest changes were observed in reproductive signaling,

GEO Accession Number	Tissue	Region / Cell Type	Microarray	SZ	HC	Description of the original study		
							associated with synaptic vesicle recycling, transmitter release and cytoskeletal dynamics	tissue remodeling, and cell differentiation. In SZ, many pathways underpinning synaptic plasticity are disrupted in both BA10 and BA22
GSE62191	Brain	Frontal cortex	Agilent 4112F	29	30	**Sample collection:**	Stanley Medical Research Institute's Neuropathology Consortium	
						**Consent / Ethics:**	The study protocol was approved by the ethics committee of A.C.Camargo Cancer Center and was performed in accordance with the Declaration of Helsinki	
						**SZ:**	Records of all patients were reviewed for DSM-IV psychiatric diagnosis independently by two senior psychiatrists	
						**HC:**	For normal controls, a structured telephone interview with a first-degree family member was carried out in all cases	
						**Exclusion criteria:**	Participants with samples with low RNA integrity were excluded	
						**Original study:**	De Baumont *et al*., Schizophrenia Research, 2015 (PMID: 25487697)	
						**Objective:**	To identify the molecular mechanisms that differentiate SZ and individuals with bipolar disorder from healthy controls	
						**Methods:**	Genome-wide expression data were used to identify co-expression of pairs of genes to assess differences between SZ and individuals with bipolar disorder. A Protein-Protein Interaction network was also used to identify additional proprieties potentially associated with the differentially expressed genes between SZ and individuals with bipolar disorder	
						**Main results:**	Co-expression analyses revealed that the pairs CCR1/SERPINA1, CCR5/HCST, C1QA/CD68, CCR5/S100A11 and SERPINA1/TLR1 present the most significant difference between SZ and individuals with BD. Moreover, network analyses showed CASP4, TYROBP, CCR1, SERPINA1, CCR5 and C1QA as having a central role in the manifestation of the disease	

GEO Accession Number	Tissue	Region / Cell Type	Microarray	SZ	HC	Description of the original study	
GSE27383	Blood	PBMCs	HG-U133_Plus_2	43	29	**Sample collection:**	Erasmus University Medical Center (EMC), Rotterdam, The Netherlands
						**Consent / Ethics:**	All subjects provided written informed consent after complete description of the study. For those patients who were too disturbed to provide consent, consent was initially given by a first-degree relative and final written consent was sought within six weeks from the patients themselves. This study was approved by the Erasmus University Medical Center Institutional Review Board and was conducted according to the declaration of Helsinki
						**SZ:**	Eligible for inclusion were male, stabilized or acutely psychotic, patients diagnosed with SZ or schizophreniform disorder according to DSM IV criteria after a Comprehensive Assessment of Symptoms and History interview (CASH) and by consensus between two senior psychiatrists. Additional criteria were recent onset (defined as duration of illness <5 years) and age (>15 and <36 years)
						**HC:**	Age-matched controls were recruited from the students and staff of the EMC medical school and hospital
						**Exclusion criteria:**	For SZ and HC, exclusion criteria were defined as follows: presence of any somatic or neurological disorders and abuse of heroin, cocaine, or alcohol. Cannabis abuse was not an exclusion criterion. Concomitant use of mood-stabilizers and/or antidepressants was an exclusion criterion. For HC, the presence of psychiatric disorders in first-degree relatives was also an exclusion criterion
						**Original study:**	van Beveren *et al.*, PLoS ONE, 2012 (PMID: 22393424)
						**Objective:**	To examine PBMCs expression levels of AKT1 in SZ versus HC, and to examine whether functional biological processes in which AKT1 plays an important role are deregulated in SZ
						**Methods:**	Genome-wide expression study in the PBMCs of SZ and HC. Functional analysis of biological processes in which AKT1 gene is involved
						**Main results:**	PBMCs expression of AKT1 was significantly decreased in SZ. 1224 genes were differentially expressed between SZ and controls. Deregulated canonical pathways were involved in cellular processes: immune system, cell adhesion and neuronal

GEO Accession Number	Tissue	Region / Cell Type	Microarray	SZ	HC	Description of the original study	
							guidance, neurotrophins and (neural) growth factors, oxidative stress and glucose metabolism, apoptosis and cell-cycle regulation. Many of these processes are associated with AKT1
GSE38484	Blood	Whole blood	HumanHT-12 V3.0	106	96	**Sample collection:**	Department of Psychiatry, University Medical Center, Utrecht, The Netherlands / Parnassia PsychoMedical Center, The Netherlands / Center for Neuropsychiatric Schizophrenia Research, Psychiatric Center Glostrup, Denmark
						**Consent / Ethics:**	The study was approved by Medical Research Ethics Committee (METC) of the University Medical Center Utrecht, The Netherlands and the Committees on Biomedical Research Ethics for the Capital Region of Denmark. All participants gave written informed consent
						**SZ:**	Psychiatric diagnoses of SZ were made according to DSM-IV-TR criteria by trained clinicians using Standardized Psychiatric interviews either The Comprehensive Assessment of Symptoms and History (CASH) or the Composite international diagnostic interview (CIDI)
						**HC:**	Unaffected controls
						**Original study:**	de Jong *et al.*, PLoS ONE, 2012 (PMID: 22761806)
						**Objective:**	To identify SZ-associated gene co-expression modules in the whole blood of SZ compared to HC
						**Methods:**	Genome-wide expression profiling from whole blood of 106 SZ and 96 HC. SZ-associated gene co-expression modules
						**Main results:**	Identification of 12 large gene co-expression modules associated with SZ. Two of the SZ-associated modules were replicated in an independent second dataset involving antipsychotic-free SZ and HC. One of these SZ-associated modules is significantly enriched with brain-expressed genes and with genetic risk variants for SZ, the hub gene in this module (ABCF1) is located in and regulated by the MHC-complex

GEO Accession Number	Tissue	Region / Cell Type	Microarray	SZ	HC	Description of the original study	
GSE62333	Skin	Fibroblasts	HuGene-1_1-st	20	20	**Sample collection:**	Samples from Brescia and Verona, Italy
						**Consent / Ethics:**	The project was approved by the local ethics committee. Written informed consent was obtained from the patients and controls. In the case of patients with a compromised ability to provide authorization, informed consent was signed by the legally authorized representative
						**SZ:**	All SZ satisfied the DSM-IV criteria for SZ. Diagnoses were confirmed using the Structured Clinical Interview for DSM-IV Axis I Disorders (SCID-I) diagnostic scale
						**HC:**	Unrelated healthy volunteers were screened for DSM-IV Axis I disorders by expert psychologists using the Mini-International Neuropsychiatric Interview (M.I.N.I.). Only healthy volunteers without a history of drug or alcohol abuse or dependence and without a personal or first-degree family history of psychiatric disorders were enrolled in the study
						**Exclusion criteria:**	For SZ and HC, exclusion criteria were defined as follows: mental retardation or cognitive disorder; serious somatic illnesses; uncorrected hypothyroidism or hyperthyroidism; age <18 and >70 years; metabolic disorders (diabetes); specific dermal diseases (e.g., dermal cancer or psoriasis). For HC, the presence of a history of drug, alcohol abuse or dependence, the presence of psychiatric disorders in first-degree relatives were additional exclusion criteria
						**Original study:**	Cattane *et al.*, PLoS ONE, 2015 (PMID: 25658856)
						**Objective:**	Differential expression analysis in skin fibroblasts of SZ and HC
						**Methods:**	Genome-wide expression study comparing skin fibroblast transcriptomic profiles from 20 SZ and 20 HC
						**Main results:**	Six genes (JUN, HIST2H2BE, FOSB, FOS, EGR1, TCF4) were strongly and significantly upregulated at the genome-wide level and confirmed by RT-PCR in SZ compared to HC

Abbreviations: GEO, Gene Expression Omnibus; SZ, individuals with schizophrenia; HC, healthy controls; DLPFC, dorsolateral prefrontal cortex; PBMCs, peripheral blood mononuclear cells; BA, Brodmann Area

Supplementary Table 3. Genes with altered expression in the postmortem brain samples of individuals with schizophrenia compared with healthy controls

**Gene**	Cerebellum		Associative striatum		Hippocampus		Parietal cortex	
	Fold Change[1]	Adjusted p-value[2]	Fold Change[1]	Adjusted p-value[2]	Fold Change[1]	Adjusted p-value[2]	Fold Change[1]	Adjusted p-value[2]
AIF1	0.86	0.02	0.79	0.03	0.99	NS	0.90	NS
CD68	0.92	0.048	1.06	NS	1.11	NS	0.95	NS
CSF1R	0.90	0.025	0.89	NS	0.86	NS	0.91	NS
CX3CR1	0.92	NS	0.62	0.03	0.50	0.01	0.82	NS
ITGAX	0.94	0.03	0.95	NS	0.86	NS	0.92	NS
IRF8	0.85	0.03	0.92	NS	1.00	NS	0.91	NS
OLR1	0.90	NS	0.63	0.03	0.72	NS	0.74	0.01

Abbreviations: NS, not significant
[1]Fold change represents the expression of the target gene in individuals with schizophrenia relative to that in healthy controls
[2]Significance of adjusted p-values set at 0.05

Supplementary Table 4. Results of ANCOVA and Bayesian analyses in the postmortem brain samples of individuals with schizophrenia compared with healthy controls

**Gene**	Cerebellum				Associative striatum				Hippocampus				Parietal cortex			
	ANCOVA		Bayesian analyses		ANCOVA		Bayesian analyses		ANCOVA		Bayesian analyses		ANCOVA		Bayesian analyses	
	F-statistic	p-value	BF_{10}	BF_{01}	F-statistic	p-value	BF_{10}	BF_{01}	F-statistic	p-value	BF_{10}	BF_{01}	F-statistic	p-value	BF_{10}	BF_{01}
AIF1	9.94	0.002	20.76	0.05	0.37	NS	0.35	2.89	/	/	/	/	/	/	/	/
CD68	5.26	0.024	3.66	0.27	/	/	/	/	/	/	/	/	/	/	/	/
CSF1R	7.02	0.010	8.14	0.12	/	/	/	/	/	/	/	/	/	/	/	/
CX3CR1	/	/	/	/	7.47	0.011	5.80	0.172	5.99	0.021	138.73	0.007	/	/	/	/
ITGAX	6.18	0.015	3.57	0.28	/	/	/	/	/	/	/	/	/	/	/	/
IRF8	5.04	0.027	5.38	0.19	/	/	/	/	/	/	/	/	/	/	/	/
OLR1	/	/	/	/	7.49	0.010	12.37	0.08	/	/	/	/	16.89	<0.001	531.55	0.002

Abbreviations: BF_{10}, Bayes Factor quantifying the evidence in favor of H_1 compared with H_0; BF_{01}, Bayes Factor quantifying the evidence in favor of H_0 compared with H_1; NS, not significant

Supplementary Table 5. Genes with altered expression in the peripheral tissue samples of individuals with schizophrenia compared with healthy controls

**Gene**	Whole blood	
	Fold Change[1]	Adjusted p-value[2]
AIF1	1.14	0.004
CD68	0.87	0.002
CSF1R	0.85	0.002
CX3CR1	0.84	0.002
ITGAX	0.91	0.040
IRF8	1.12	0.004
OLR1	1.1	0.002
TLR2	1.1	0.002

[1]Fold change represents the expression of the target gene in individuals with schizophrenia relative to that in healthy controls
[2]Significance of adjusted p-values set at 0.05

Supplementary Table 6. Results of ANCOVA and Bayesian analyses in the peripheral tissue samples of individuals with schizophrenia compared with healthy controls

**Gene**	Whole blood			
	ANCOVA		Bayesian analyses	
	F-statistic	p-value	BF_{10}	BF_{01}
AIF1	10.01	0.002	9.93	0.1
CD68	11.88	<0.001	43.84	0.02
CSF1R	22.48	<0.001	355.9	0.003
CX3CR1	37.42	<0.001	98837	0.00001
ITGAX	3.41	NS	1.33	0.76
IRF8	6.86	0.009	10.2	0.1
OLR1	15.19	<0.001	316.1	0.003
TLR2	16.43	<0.001	26.5	0.04

Abbreviations: BF_{10}, Bayes Factor quantifying the evidence in favor of H_1 compared with H_0; BF_{01}, Bayes Factor quantifying the evidence in favor of H_0 compared with H_1; NS, not significant

References

1. Patir A, Shih B, McColl BW, Freeman TC. A core transcriptional signature of human microglia: Derivation and utility in describing region-dependent alterations associated with Alzheimer's disease. Glia. 2019;67:1240–1253.
2. Snijders GJLJ, van Zuiden W, Sneeboer MAM, Berdenis van Berlekom A, van der Geest AT, Schnieder T, et al. A loss of mature microglial markers without immune activation in schizophrenia. Glia. 2021;69:1251–1267.
3. Gandal MJ, Haney JR, Parikshak NN, Leppa V, Ramaswami G, Hartl C, et al. Shared molecular neuropathology across major psychiatric disorders parallels polygenic overlap. Science. 2018;359:693–697.
4. Bergon A, Belzeaux R, Comte M, Pelletier F, Hervé M, Gardiner EJ, et al. CX3CR1 is dysregulated in blood and brain from schizophrenia patients. Schizophr Res. 2015;168:434–443.
5. Sneeboer MAM, van der Doef T, Litjens M, Psy NBB, Melief J, Hol EM, et al. Microglial activation in schizophrenia: Is translocator 18 kDa protein (TSPO) the right marker? Schizophr Res. 2020;215:167–172.
6. Zhu Y, Webster MJ, Murphy CE, Middleton FA, Massa PT, Liu C, et al. Distinct Phenotypes of Inflammation Associated Macrophages and Microglia in the Prefrontal Cortex Schizophrenia Compared to Controls. Front Neurosci. 2022;16.
7. Patel T, Carnwath TP, Wang X, Allen M, Lincoln SJ, Lewis-Tuffin LJ, et al. Transcriptional landscape of human microglia implicates age, sex, and APOE-related immunometabolic pathway perturbations. Aging Cell. 2022;21:e13606.
8. Narayan S, Tang B, Head SR, Gilmartin TJ, Sutcliffe JG, Dean B, et al. Molecular profiles of schizophrenia in the CNS at different stages of illness. Brain Res. 2008;1239:235–248.
9. de Jong S, Boks MPM, Fuller TF, Strengman E, Janson E, de Kovel CGF, et al. A gene co-expression network in whole blood of schizophrenia patients is independent of antipsychotic-use and enriched for brain-expressed genes. PloS One. 2012;7:e39498.
10. de Baumont A, Maschietto M, Lima L, Carraro DM, Olivieri EH, Fiorini A, et al. Innate immune response is differentially dysregulated between bipolar disease and schizophrenia. Schizophr Res. 2015;161:215–221.
11. Arion D, Corradi JP, Tang S, Datta D, Boothe F, He A, et al. Distinctive transcriptome alterations of prefrontal pyramidal neurons in schizophrenia and schizoaffective disorder. Mol Psychiatry. 2015;20:1397–1405.
12. Horiuchi Y, Kondo MA, Okada K, Takayanagi Y, Tanaka T, Ho T, et al. Molecular signatures associated with cognitive deficits in schizophrenia: a study of biopsied olfactory neural epithelium. Transl Psychiatry. 2016;6:e915–e915.
13. Jeffreys SH. The Theory of Probability. Third Edition. Oxford, New York: Oxford University Press; 1998.
14. Maycox PR, Kelly F, Taylor A, Bates S, Reid J, Logendra R, et al. Analysis of gene expression in two large schizophrenia cohorts identifies multiple changes associated with nerve terminal function. Mol Psychiatry. 2009;14:1083–1094.

15. Chen C, Cheng L, Grennan K, Pibiri F, Zhang C, Badner JA, et al. Two gene co-expression modules differentiate psychotics and controls. Mol Psychiatry. 2013;18:1308–1314.
16. Lanz TA, Reinhart V, Sheehan MJ, Rizzo SJS, Bove SE, James LC, et al. Postmortem transcriptional profiling reveals widespread increase in inflammation in schizophrenia: a comparison of prefrontal cortex, striatum, and hippocampus among matched tetrads of controls with subjects diagnosed with schizophrenia, bipolar or major depressive disorder. Transl Psychiatry. 2019;9:151.
17. Barnes MR, Huxley-Jones J, Maycox PR, Lennon M, Thornber A, Kelly F, et al. Transcription and pathway analysis of the superior temporal cortex and anterior prefrontal cortex in schizophrenia. J Neurosci Res. 2011;89:1218–1227.
18. van Beveren NJM, Buitendijk GHS, Swagemakers S, Krab LC, Röder C, de Haan L, et al. Marked reduction of AKT1 expression and deregulation of AKT1-associated pathways in peripheral blood mononuclear cells of schizophrenia patients. PloS One. 2012;7:e32618.
19. Cattane N, Minelli A, Milanesi E, Maj C, Bignotti S, Bortolomasi M, et al. Altered Gene Expression in Schizophrenia: Findings from Transcriptional Signatures in Fibroblasts and Blood. PLOS ONE. 2015;10:e0116686.
